# Correction to ‘A possible case of inverted lifestyle in a new bivalved arthropod from the Burgess Shale’

**DOI:** 10.1098/rsos.192111

**Published:** 2020-01-08

**Authors:** Alejandro Izquierdo-López, Jean-Bernard Caron

*R. Soc. open sci.* **6**, 191350. (Published 13 November 2019). (doi:10.1098/rsos.191350)

This correction is issued to amend the version of record of ‘A possible case of inverted lifestyle in a new bivalved arthropod from the Burgess Shale’ published in *Royal Society Open Science*. There were a number of textual and typographical changes to the earlier version of the published article text. The journal is issuing this Correction to provide the correct version of the article, which appears below.
