# Supplementary material for: A possible case of inverted lifestyle in a new bivalved arthropod from the Burgess Shale
Source: R Soc Open Sci. 2019 Nov 13;6(11):191350. doi: 10.1098/rsos.191350 (PMC6894550; doi:10.1098/rsos.191350)
Supplement: Supplementary Material [file rsos191350supp1.docx]

**Supplementary Material**

1. **Specimens of *Fibulacaris nereidis* deposited at the Royal Ontario Museum and used in this study.** Locality: WHY-NW) Mount Whymper, Kootenay National Park, British Columbia, Canada; MC) Marvel Canyon, Kootenay National Park, British Columbia, Canada; TOK) Tokumm Creek, Kootenay National Park, British Columbia, Canada. The complete specimens of *Fibulacaris nereidis* can be found in the Supplementary Documents.

| **ROMIP No.** | **Field**  **No.** | **Part & Counter-part** | **Locality** | **Stratigraphy** | **Preservation**  **Details** | **Size: Length and Height (mm)/**  **Figure** |
| --- | --- | --- | --- | --- | --- | --- |
| 65354 | 2014-29 | NC | WHY-NW | Talus | Just carapace | 9 L; 4 H  Not figured |
| 65355 | 2014-279 | NC | WHY-NW | Talus | Just carapace | 15 L; 4 H  Fig 5; B |
| 65356 | 2014-387 | AB | WHY-NW | Talus | Just carapace | 15 L; 7 H  Not figured |
|  |  |  |  |  | Longest spine |  |
| 65357 | 2014-1417 | AB | MC | -349 cm | Broken carapace, Legs | 12 L; 3 H  Not figured |
| 65358 | 2014-1418 | NC | MC | -347 cm | Just carapace | 10 L; 3.5 H  Not figured |
| 65359 | 2014-1465 | AB | MC | -374 cm | Ventral view | Not measured  Fig 3; A,B,C,D,E,F,G |
| 65360 | 2015-380 | NC | TOK | Talus | Weathered, carapace and legs | 10 L; 4 H  Not figured |
| 65361 | 2016-559 | NC | TOK | Talus | Carapace, legs | 5 L; 1 H  Not figured |
| 65362 | 2016-567 | NC | TOK | Talus | Just carapace | 12 L; 7 H  Not figured |
| 64511 | 2016-574 | AB | TOK | Talus | Just carapace | Not measured  Fig 5; K |
| 65363 | 2016-631 | NC | TOK | Talus | Weathered  carapace, body, gut | 20 L; 6 H  Fig 4; B  Supp.F.1; B, C, D |
| 65364 | 2018-50 | AB | TOK | In-situ | Just carapace, small, traces eyes | 12 L; 2 H  Not figured |
| 65365 | 2018-52 | NC | TOK | In-situ | Just carapace | 6 L; 2 H  Not figured |
| 65366 | 2018-55 | AB | TOK | In-situ | Carapace, eye, broken spine | 14 L; 4 H  Not figured |
| 65367 | 2018-64 | AB | TOK | In-situ | Carapace, body, small | 6 L; 2 H  Not figured |
| 65368 | 2018-87 | NC | TOK | In-situ | Just carapace | 10 L; 2.5 H  Fig 5; J |
| 65369 | 2018-88 | AB | TOK | In-situ | Carapace, gut | 10 L; 4 H  Fig 4; A |
| 65370 | 2018-91 | AB | TOK | In-situ | Just carapace | 16 L; 6 H  Not figured |
| 65371 | 2018-117 | AB | TOK | In-situ | Just carapace | 19 L; 9 H  Not figured |
| 65372 | 2018-118 | AB | TOK | In-situ | Just carapace | 14 L; 5 H  Not figured |
| 65373 | 2018-125 | NC | TOK | In-situ | Just carapace, eye | 15 L; 4.5 H  Not figured |
| 65374 | 2018-127 | NC | TOK | In-situ | Just carapace | 16 L; 7 H  Not figured |
| 65375 | 2018-150 | AB | TOK | In-situ | Just carapace | 5 L; 2.5 H  Not figured |
| 65376 | 2018-182 | NC | TOK | In-situ | Weathered, just carapace | 14 L; 7 H  Not figured |
| 65377 | 2018-183 | AB | TOK | In-situ | Just carapace | 14 L; 4 H  Not figured |
| 65378 | 2018-198 | AB | TOK | In-situ | Just carapace | 19 L; 8 H  Not figured |
| 65379 | 2018-280 | AB | TOK | In-situ | Carapace, body detached | 10 L; 4 H  Fig 5; E, G |
| 65380 | 2018-283 | AB | TOK | In-situ | Holotype,  Carapace, body, main features | 16 L; 5 H  Fig 1; A,B (drawing) |
| 65381 | 2018-294 | NC | TOK | In-situ | Just carapace | 12 L; 4 H  Not figured |
| 65382 | 2018-332 | AB | TOK | Talus | Carapace, body detached | Not measured  Fig 5; F, H |
| 65383 | 2018-416 | AB | TOK | In-situ | Broken carapace, body preserved | 15 L; 10 H  Fig 5; C |
| 65384 | 2018-444 | NC | TOK | In-situ | Just carapace | 14 L; 4.5 H  Not figured |
| 65385 | 2018-446 | AB | TOK | In-situ | Carapace, part of the body, eyes | 12 L; 4 H  Fig 5; D |
| 65386 | 2018-453 | AB | TOK | Talus | Carapace, some legs | 15 L; 10 H  Not figured |
| 65387 | 2018-483 | AB | TOK | In-situ | Just carapace, eye | 17 L; 5 H  Not figured |
| 65388 | 2018-486 | AB | TOK | In-situ | Carapace broken, body | 13 L; 6 H  Fig 4; E |
| 65389 | 2018-527 | NC | TOK | In-situ | Carapace broken, part of the body | 9 L; 4 H  Not figured |
| 65390 | 2018-534 | AB | TOK | In-situ | Carapace, gut, eye | 10 L; 6 H  Fig 4; D |
| 65392 | 2018-560 | NC | TOK | In-situ | Weathered, Just carapace | 13 L; 7 H  Not figured |
| 65393 | 2018-707 | AB | TOK | In-situ | Carapace broken, part of the body | 8 L; 3 H  Not figured |
| 65394 | 2018-708 | AB | TOK | In-situ | Carapace folded, part of the body | 12 L; 3 H  Not figured |
| 65395 | 2018-734 | NC | TOK | In-situ | Carapace, body, legs | 17 L; 6 H  Fig 5; A |
| 65396 | 2018-737 | AB | TOK | In-situ | Carapace without spine | 12 L; 4 H  Not figured |
| 65397 | 2018-773 | AB | TOK | In-situ | Paratype, Carapace broken, body | 14 L; 4 H  Fig 2; A,B |
| 65398 | 2018-833 | AB | TOK | In-situ | Just carapace | 12 L; 6 H  Not figured |
| 65399 | 2018-871 | AB | TOK | In-situ | Just carapace | 14 L; 5 H  Not figured |
| 65400 | 2018-913 | AB | TOK | In-situ | Paratype, Carapace, body | 17 L; 8 H  Fig 2; E,F |
| 65401 | 2018-356_1 | AB | TOK | In-situ | Carapace, body | Not measured  Fig 5; I |
| 65401 | 2018-356_2 | AB | TOK | In-situ | Carapace, body | 14 L; 4 H  Not figured |
| 65401 | 2018-356_3 | AB | TOK | In-situ | Carapace, body | Not measured  Fig 4; F,G |
| 65455 | 2018  86 | AB | TOK | In-situ | Cluster  6 specimens | Not measured  Not figured |
| 65456 | 2018  101 | AB | TOK | In-situ | Cluster  3 specimens | Not measured  Not figured |
| 65457 | 2018  186 | AB | TOK | In-situ | Cluster  11 specimens | Not measured  Fig 4; Supp. F. 2; A, B |
| 65458 | 2018  465 | AB | TOK | In-situ | Carapace broken, legs | Not measured  Fig 2; C |
| 65459 | 2018  729 | NC | TOK | In-situ | Dorsal view,  Carap, legs | Not measured  Not figured |
| 65460 | 2018  829 | NC | TOK | In-situ | Ventral view  Carap, legs | Not measured  Fig 1; D |
| 65461 | 2018  749 | AB | TOK | In-situ | Ventral view  Carap | Not measured  Fig 1; C |
| 65462 | 2018  711 | AB | TOK | In-situ | Carapace broken, legs | Not measured  Fig 2; D |
| 65463 | 2018  230 | AB | TOK | In-situ | Carapace | 18 L; 6 W  Not figured |
| 65464 | 2018  174 | NC | TOK | In-situ | Carapace | 11 L; 4 W  Not figured |
| 65465 | 2018  36 | AB | TOK | In-situ | Carapace | 12 L; 6 W  Not figured |
| 65466 | 2018  12 | NC | TOK | Talus | Carapace | 15 L; 5 W  Not figured |
| 65467 | 2016  558 | NC | TOK | In-situ | Carapace, body | 7 L; 2 W  Not figured |

1. **New taxa considered in the dataset.**

All taxa presented in this dataset appeared previously in Vannier et al., (1). The complete analysis can be found in Supplementary Figure 3 and Supplementary Figure 4. Additional taxa include:

*Clypecaris* (2)

*Nereocaris briggsi* (3)

*Nereocaris exilis* (4)*:* present in (1)*,* but separated into *N.briggsi* and *N.exilis*

*Perspicaris:* present in (1)*,* but recoded and based on *Perspicaris recondita* (5)

*Loricicaris* (3)

*Ercaicunia* (6)

*Fibulacaris nereidis* [this article]

*Yawunik* (7)

*Ercaia* (8)

*Peronopsis* (9)

*Tegopelte* (10)

*Kodymirus* (11)

*Cyzicus:* as a representative of Spinicaudata

*Mantodea:* based on higher Mantodea (ie. Mantidae)

1. **Characters used in phylogenetic analysis. Abbreviations: [Vetal3]. Character number 3 in the matrix of Vannier et al.** (1)

**General characters:**

[1] Type of segmentation [Vetal3]

0. Sclerotized

1. Arthrodized (=tergal)

[2] Calcified cuticle [Vetal4]

0. Absent

1. Present

[3] Main calcification type [Vetal5]

0. Calcium carbonate

1. Calcium phosphate

[4] Holometaboly [Vetal6]

0. Absent

1. Present

**Lobopodian-like characters**

**Remarks:** mainly present for radiodont and *Isoxys*-only traits.

[5] External anteriorization restricted to a single pair of frontalmost appendages [Vetal7]

0. Absent

1. Present

[6] Lobopodous limbs [Vetal8]

0. Absent

1. Present

[7] Type of main lobopodous trunk limb [Vetal9]

0. Short, conical, subequal or shorter than trunk width

1. Elongated, slender, longer than trunk width

[8] Flap-like lateral limbs [Vetal10]

0. Absent

1. Present

[9] Differentiation at limb insertion [Vetal12]

0. Absent

1. Present

**Eye characters**

[10] Median eyes [Vetal 19]

0. Absent

1. Present

[11] Number of median eyes [Vetal 20]

0. 2

1. 3

2. 4

[12] Rhabdomeric lateral eye [Vetal 21]

0. Absent

1. Present

[13] Type of lateral eyes [Vetal 22]

0. Simple lens with cup-shaped retina

1. Faceted (compound)

2. Stemmata

[14] Type of corneagenous cells [Vetal 23]

0. Many

1. Two

[15] Tetraconate condition [Vetal 24]

0. Absent

1. Present

[16] Number of nested optic neuropils [Vetal 25]

0. 1

1. 2

2. 3

[17] Multi-layered rhabdomeres [Vetal 26]

0. Absent

1. Present

[18] Eyes embedded within tergal shield [Vetal 27]

0. Absent

1. Present

[19] Opthalmic ridges [Vetal 28]

0. Absent

1. Present

[20] Lateral eyes pedunculate [Vetal 29]

0. Absent

1. Present

[21] Pedunculate eyes large and ovate, part of a prominent ocular segment projecting anteriad [Vetal 30]

0. Absent

1. Present

**Cephalic characters**

[22] Somital head (as tagma I) defined by series of appendages and/or external segmentation [Vetal 31]

0. Absent

1. Present

[23] Somites defining anteriormost tagma [Vetal 32]

0. 5

1. 6

2. 7

3. 8

[24] Tergite of the ocular (protocerebral) somite [Vetal 33]

0. Absent

1. Present

[25] Tergite of the ocular (protocerebral) somite, type [Vetal 34]

0. Rounded

1. Sub-triangular

[26] Tergal sclerotization of the post-ocular somite [Vetal 35]

0. Absent

1. Present

[27] Tergal sclerotization type [Vetal 36]

0. Tergites with posterior expansion, some cephalic tergites can be freely articulating (carapace)

1. Tergites with limited expansion, cephalic tergites fused (shield)

[28] Carapacal valves [Vetal 37]

0. Bivalved

1. Fused

[29] Bivalved carapace type [Vetal 38]

0. Type I: Sub-straight cross-section, covering body similar to head shield

1. Type II: Convex cross-section, enveloping body laterally

[30] Type II bivalved configuration [Vetal 39]

0.Unfused along most of dorsal margin

1.Fused along most of dorsal margin

[31] Covering of the type II bivalved carapace (when body fully extended antero-posteriorly) [Vetal 40]

0. At least two thirds of body length

1. Cephalothorax

[32] Articulation of posterior margin of shield with first trunk segment [Vetal 41]

0. Tergal overlap

1. Closure

[33] Segmental impression in shield [Vetal 42]

0. Absent

1. Present

[34] Occipital lobe [Vetal 43]

0. Absent

1. Present

[35] Pair of occipital carinae [Vetal 44]

0. Absent

1. Present

[36] Anterior reduction of segments and/or appendages [Vetal 45]

0. Absent

1. Present

[37] Compaction of the cephalic unit [Vetal 46]

0. Absent

1. Present

[38] Doublure [Vetal 47]

0. Absent

1. Present

[39] Cephalic kinesis [Vetal 48]

0. Absent

1. Present

**Brain characters**

[40] Ganglia of post-oral appendages fused into single nerve mass [Vetal 49]

0. Absent

1. Present

[41] Contiguity of the first two post-protocerebral ganglia [Vetal 50]

0. Absent

1. Present

[42] Fan-shaped body in brain [Vetal 51]

0. Absent

1. Present

[43] Position of midline neuropil [Vetal 52]

0. Superficial to protocerebrum

1. Embedded within protocerebral matrix

[44] Olfactory lobes linked to a lateral component of protocerebrum by olfactory globular tract [Vetal 53]

0. Absent

1. Present

[45] Deutocerebral olfactory lobe with glomeruli [Vetal 54]

0. Absent

1. Present

[46] Protocerebral bridge [A&C 53]

0. Absent

1. Present

**Sternites (Cephalon)**

[47] Sternites [Vetal 56]

0. Absent

1. Present

[48] Endosternum [Vetal 57]

0. Absent

1. Present

[49] Labrum [Vetal 58]

0. Absent

1. Present

[50] Labrum expression and location [Vetal 59]

0. Expressed anteriormost or not distinct

1. Expressed as ventral pre-oral structure

[51] Type of ventral labrum [Vetal 60]

0. Single fleshy protrusion

1. Plate with underlying soft tissues

[52] Fusion of pre-oral structures [Vetal 61]

0. Absent

1. Present

[53] Hypostome [Vetal 62]

0. Absent

1. Present

[54] Hypostome type [Vetal 63]

0. Conterminant

1. Natant

[55] Hypostome accommodating antennules and extensively covering the mouth [Vetal 64]

0. Absent

1. Present

[56] Labium [Vetal 65]

0. Absent

1. Present

[57] Post-hypostomal sternites externally developed within segments 2–4 [Vetal 66]

0. Absent

1. Present

[58] Fusion of post-hypostomal sternites externally developed within segments 2–4 [Vetal 67]

0. Absent

1. Present

[59] Metastoma [Vetal 68]

0. Absent

1. Present

[60] Coxosternite [Vetal 69]

0. Absent

1. Present

[61] Both larval and imaginal head has tendency to form a hypostomal bridge [Vetal 70]

0. Absent

1. Present

**Frontalmost appendages**

[62] Arthrodization of first axial appendage [Vetal71]

0. Absent

1. Present

[63] Ocular (=peduncular) lobes [Vetal72]

0. Absent

1. Present

[64] Bipartite frontalmost ventral protrusion [Vetal73]

0. Absent

1. Present

[65] Branching frontalmost appendage [Vetal74]

0. Absent

1. Present

[66] Rami of branching frontalmost appendage originating from different podomeres [Vetal75]

0. Absent

1. Present

[67] Frontalmost appendage with flagellate extensions [Vetal76]

0. Absent

1. Present

[68] Frontalmost appendage a chelicera, i.e. chelate or subchelate with only two opposing faces [Vetal77]

0. Absent

1. Present

[69] Orientation of first axial appendage [Vetal78]

0. Ventro-frontal

1. Dorsal

[70] Segmentation of frontalmost arthrodized appendage [Vetal79]

0. Multi-segmented

1. Reduced

[71] Arthrodized frontalmost appendage, multi-segmented type [Vetal80]

0. Robust, thick branch

1. Long antennular

[72] Inner (ventral) spinose outgrowths on arthrodized frontalmost appendage [Vetal81]

0. Absent

1. Present

[73] Type of inner (ventral) spinose outgrowths on frontalmost appendage [Vetal82]

0. Sub-equal length or tapering gradually along entire margin

1. Elongate mid-margin

[74] Secondary spines on inner (ventral) spinose outgrowths of frontalmost appendage [Vetal83]

0. Absent

1. Present

[75] Outer (dorsal) spinose outgrowths on arthrodized frontalmost appendage [Vetal84]

0. Absent

1. Present

[76] Outer (dorsal) spinose outgrowth on arthrodized frontalmost appendage with elongate terminal spine [Vetal85]

0. Absent

1. Present

**Other cephalic limbs**

[77] All cephalic endopods posterior to second pair well-developed [Vetal86]

0. Absent

1. Present

[78] Endopod of second appendage pair [Vetal87]

0. Developed

1. Reduced

[79] Endopod of third appendage pair [Vetal88]

0. Developed

1. Reduced

[80] Endopod of fourth appendage pair [Vetal89]

0. Developed

1. Reduced

[81] Exopod of fourth appendage pair [Vetal90]

0. Developed

1. Reduced

[82] Endopod of fifth appendage pair [Vetal91]

0. Developed

1. Reduced

[83] Some cephalic endopods are walking limbs [Vetal92]

0. Absent

1. Present

[84] Repeated appendage morphology in tagma I [Vetal93]

0. Absent

1. Present

[85] Dichotomy in appendage morphology between tagma I and tagma II [Vetal94]

0. Absent

1. Present

[86] Proximo-distal differentiation of endopod podomeres in head (tagma I) [Vetal95]

0. Absent

1. Present

[87] Podomere number in head (tagma I) [Vetal96]

0. 7

1. <7

2. >7

[88] Post-antennular appendage expressed [Vetal97]

0. Absent

1. Present

[89] Post-antennular appendage differentiated [Vetal98]

0. Absent

1. Present

[90] Chelate or sub-chelate termination of post-antennular appendage [Vetal99]

0. Absent

1. Present

[91] Ramification of post-antennular appendage [Vetal100]

0. Uniramous

1. Biramous

[92] Developed endites on endopod of post-antennular appendage [Vetal101]

0. Absent

1. Present

[93] Endopod of post-antennular appendage annulate or flagellate [Vetal102]

0. Absent

1. Present

[94] Podomere number of endopod of post-antennular appendage [Vetal103]

0. <7

1. 7

[95] Coxa on post-antennular appendage [Vetal104]

0. Absent

1. Present

[96] Exopod of post-antennular appendage, type [Vetal105]

0. Stenopodous

1. Annulate

2. Rod-shaped

3. Paddle

4. Tripartite

[97] Exopods on cephalic appendages excluding two anteriormost pairs [Vetal106]

0. Absent

1. Present

[98] Exopod of cephalic appendages excluding two anteriormost pairs, type [Vetal107]

0. Stenopodous

1. Annulate

2. Rodiform

3. Paddle

4. Tripartite

[99] Multisetose, rounded tip on cephalic exopods [Vetal108]

0. Absent

1. Present

[100] Partial detachment of exopods from main limb branch in head tagma [Vetal109]

0. Absent

1. Present

[101] Enditic outgrowths on cephalic endopods excluding two anteriormost pairs [Vetal110]

0. Absent

1. Present

[102] Endopod of third cephalic appendage chelate or subchelate [Vetal111]

0. Absent

1. Present

[103] Third cephalic appendage with a well-developed gnathobase [Vetal112]

0. Absent

1. Present

[104] Third cephalic appendage a mandible [Vetal113]

0. Absent

1. Present

[105] Mandible with three-segmented endopod, appressed on the ventral side of the head, curving inward [Vetal114]

0. Absent

1. Present

[106] Mandibular palp [Vetal115]

0. Non-developed

1. Developed

[107] Telognathic mandible [Vetal116]

0. Absent

1. Present

[108] Mandibular gnathal edge [Vetal117]

0. Consisting of molar and incisor process

1. Only ellipsoid pars molaris present

2. Row of parallel teeth

3. Shovel with terminal teeth

4. Group of paired teeth and hair pad

[109] Mandibular lamellate combs [Vetal118]

0. Absent

1. Present

[110] Hypopharynx [Vetal119]

0. Absent

1. Present

[111] Modified endopod/palp on fourth cephalic appendage [Vetal120]

0. Absent

1. Present

[112] Modified endopod/palp on fourth cephalic appendage, type [Vetal121]

0. Reduced, vestigial, undeveloped

1. Well developed

[113] Post-mandibular plate formed by the fusion of the maxilla and the intermaxillary sternum [Vetal122]

0. Absent

1. Present

[114] Cephalic appendages 4 and 5 ending with chelate termination [Vetal123]

0. Absent

1. Present

[115] Fifth cephalic appendage [Vetal124]

0. Integrated to gnathal plate (labium)

1. Reduced, enditic

[116] Fifth cephalic appendage vestigial [Vetal125]

0. Absent

1. Present

[117] Fifth cephalic appendage with developed palp [Vetal126]

0. Absent

1. Present

**Mouth and stomodaeal area**

[118] Internalization of mouthparts [Vetal127]

0. Absent

1. Present

[119] Oral cone [Vetal128]

0. Absent

1. Present

[120] Atrium oris [Vetal129]

0. Absent

1. Present

[121] Type of circumoral structures [Vetal131]

0. Toothed lips

1. Lamellae

2. Plates

3. Hypostome-labrum complex

[122] Circumoral structures sclerotized [Vetal132]

0. Absent

1. Present

[123] Proboscis [Vetal133]

0. Absent

1. Present

**Alimentary tract and other internal characters**

[124] Stomach [Vetal134]

0. Absent

1. Present

[125] Stomach in a frontal position [Vetal135]

0. Absent

1. Present

[126] Stomach—additional pouch [Vetal136]

0. Absent

1. Present

[127] Secondary organs connected to the central digestive duct [Vetal137]

0. Absent

1. Present

[128] Secondary digestive organs serially repeated along the post-cephalic portion of the gut [Vetal138]

0. Absent

1. Present

[129] Shape of post-cephalic secondary digestive structures [Vetal139]

0. Reniform

1. Bulgy triangles

2. Caeca

[130] Striations on post-cephalic secondary digestive structures [Vetal140]

0. Absent

1. Present

[131] Branching of post-cephalic secondary digestive structures [Vetal141]

0. Absent

1. Present

[132] Differentiation of cephalic secondary digestive structures (compared to trunk) [Vetal142]

0. Absent

1. Present

[133] Ramification of cephalic secondary digestive structures [Vetal143]

0. Absent

1. Present

[134] Branching of cephalic secondary digestive structures [Vetal144]

0. Absent

1. Present

[135] Peritrophic membrane [Vetal145]

0. Absent

1. Present

[136] Metameric ganglia on nerve cord [Vetal146]

0. Absent

1. Present

[137] Metanephridia with sacculus containing podocytes [Vetal147]

0. Absent

1. Present

[138] Segmental invaginations of neuroectoderm giving rise to ventral organs [Vetal148]

0. Absent

1. Present

**Trunk**

[139] Thorax [Vetal149]

0. Absent

1. Present

[140] Number of thoracic somites [Vetal150]

0. 11

1. 5

2. 8

3. 3

[141] Abdomen [Vetal151]

0. Absent

1. Present

[142] Number of core trunk segments [Vetal152]

0. >14

1. 12-14

2. 9

3. 7-8

4. <7

[143] Seventh appendage integrated into the prosoma [Vetal153]

0. Absent

1. Present

[144] Tergite of eighth somite (counting the ocular somite as the first) drastically reduced as a “microtergite” [Vetal154]

0. Absent

1. Present

[145] Post-cephalic appendages covered by sclerotic plates (opercula) [Vetal155]

0. Absent

1. Present

[146] Multisegmentation [Vetal156]

0. Absent

1. Present

[147] Tergo-sternal decoupling [Vetal157]

0. Absent

1. Present

[148] Tergo-sternal decoupling, type [Vetal158]

0. Polypody

1. Polypody and “polysternity”

2. “Polytergity” (autapomorphy of symphylan myriapods)

[149] Pleurae [Vetal159]

0. Reduced or fused

1. Developed

[150] Tergo-pleural rings [Vetal160]

0. Absent

1. Present

[151] Pleural orientation [Vetal161]

0. Horizontal

1. Around body

[152] Pleural length [Vetal162]

0. Short, i.e. equal or inferior to body diameter

1. Long, i.e. exceeding body diameter

[153] Articulating ridge [Vetal163]

0. Absent

1. Present

[154] Articulating ridge, type [Vetal164]

0. Single

1. Antero-posterior

[155] Transverse stipital muscle [Vetal165]

0. Absent

1. Present

**Trunk appendages and other appendicular characters**

[156] Proximo-distal differentiation of endopod podomeres in tagma II [Vetal166]

0. Absent

1. Present

[157] Podomere number in tagma II [Vetal167]

0. 7

1. <7

2. >7

[158] Maxillipeds [Vetal168]

0. Absent

1. Present

[159] Tergites of maxilliped segments fused to head shield [Vetal169]

0. Absent

1. Present

[160] Single main maxilliped [Vetal170]

0. Absent

1. Present

[161] Slit sensilla [Vetal171]

0. Absent

1. Present

[162] Basis (basipod) [Vetal172]

0. Absent

1. Present

[163] Basipod formed of at least two elements [Vetal173]

0. Absent

1. Present

[164] Basipod multi-segmented [Vetal174]

0. Absent

1. Present

[165] Multiple endites on basipod [Vetal175]

0. Absent

1. Present

[166] Proximal endite [Vetal176]

0. Absent

1. Present

[167] Coxa as entire pre-basal podomere [Vetal177]

0. Absent

1. Present

[168] Precoxa as whole pre-coxal podomere [Vetal178]

0. Absent

1. Present

[169] Pleurites formed by several sclerotic elements surrounding limb insertion [Vetal179]

0. Absent

1. Present

[170] Arrangement of pleurites [Vetal180]

0. Outer/proximal and distal/inner sets

1. Multiple sclerotic pieces

[171] Gnathobases [Vetal181]

0. Absent

1. Present

[172] One or more gnathobase(s) reduced in tagma I [Vetal182]

0. Absent

1. Present

[173] Secondary appendicular outgrowths on trunk [Vetal183]

0. Absent

1. Present

[174] Secondary appendicular outgrowths on trunk, type [Vetal184]

0. Lobopodous

1. Sclerotized

[175] Proximal lamellae [Vetal185]

0. Absent

1. Present

[176] Proximal lamellae internalized [Vetal186]

0. Absent

1. Present

[177] Trunk endopod reduced posterior to head tagma [Vetal187]

0. Absent

1. Present

[178] Limb arthrodization in trunk [Vetal188]

0. Absent

1. Present

[179] Trunk exopod posterior to head tagma, type [Vetal189]

0. Paddle/lobe

1. Rodiform

2. Annulate

3. Reduced

4. Three-segmented

5. Phyllopodous

[180] Endopod strongly developed in thorax (or anterior trunk if thorax undifferentiated) [Vetal190]

0. Absent

1. Present

[181] Phyllopodous-type limbs anywhere on body [Vetal191]

0. Absent

1. Present

[182] Terminal endopods stenopodous [Vetal192]

0. Absent

1. Present

[183] Identical morphology of endopod and exopod rami on pleopods/post-thorax [Vetal193]

0. Absent

1. Present

[184] Annulation of at least one pair of exopods [Vetal194]

0. Absent

1. Present

[185] Subdivision of at least one pair of exopods [Vetal195]

0. Absent

1. Present

[186] Subdivision of at least one pair of exopods, type [Vetal196]

0. Bipartite

1. Tripartite

[187] Attachment segment in lobate exopod [Vetal197]

0. Absent

1. Present

[188] Epipod [Vetal198]

0. Absent

1. Present

[189] Endite as a latero-distal projection on endopod podomeres [Vetal199]

0. Absent

1. Present

[190] Pusher legs with paddle tips [Vetal200]

0. Absent

1. Present

[191] Developed endites on endopod podomeres in trunk (tagma II and III) [Vetal201]

0. Absent

1. Present

[192] Paired spines on endopod podomere [Vetal202]

0. Absent

1. Present

[193] Short spines on endopod podomere [Vetal203]

0. Absent

1. Present

[194] Multiple setae on endopod podomere [Vetal204]

0. Absent

1. Present

[195] Paired elongate spines distally on endopod [Vetal205]

0. Absent

1. Present

[196] Limb tip [Vetal206]

0. Pad

1. Juxtaposed claws

2. Trident of claws

3. Chelate or sub-chelate

4. Double claw

5. Multiple spines

6. Single claw

**Telson**

[197] Sclerotization of termination [Vetal207]

0. Absent

1. Present [198] Telson developed [Vetal208]

0. Absent

1. Present

[199] Telson type [Vetal209]

0. Spine

1. Plate

2. Spatula

[200] Anus location [Vetal210]

0. Terminal, last segment

1. Base of telson

[201] Caudal rami [Vetal211]

0. Absent

1. Present

[202] Additional caudal processes [Vetal212]

0. Absent

1. Present

[203] Furca [Vetal213]

0. Absent

1. Present

[204] Uropods sensu stricto [Vetal214]

0. Absent

1. Present

[205] Caudal rami, type

0. Spinose

1. Paddle like

2. Cerci-like

3. Segment-like

4. Plate-like

5. Claw-like

**Remarks**: This character expands on Vannier et al., (1) [Character 215]. It includes annulate, multisegmented structures (Cerci-like) as in *Tokummia* (12), bunt segment-like structures as in *Bredocaris* (segment like) (13), abdominal/telsonic horizontal plates that can cover the anus, such as in *Kuamaia* (14) and telsons that directly end in terminal spines, such as in the cercopods in Spinicaudata.

[206] Caudal rami, number of segments

1. No segmentation
2. Tri-segmented
3. Multisegmented

**Remarks:** Caudal rami can be unsegmented or present certain segmentation, which may recover some relations across Cambrian “bivalved arthropods”. Tri-segmented caudal rami, for example, only apply for two Cambrian species with unique caudal rami (ie. *Waptia*, *Nereocaris exilis*), divided in three parts of equal size (1,4).

[207] Caudal rami, spines

0. Absent

1. Present

**Remarks**: Some species present spines on the caudal rami (ie. some Leptostraca, *Perspicaris*). This character may be highly plastic, but could help recover affinities between fossil species.

[208] Caudal rami, setae

0. Absent

1. Present

**Remarks**: Some species present setae on their caudal rami (e.g. in some Copepoda), potentially implying a sensorial or other alternative function.

[209] Ovipositor

0. Absent

1. Present

2. Reduced

**Remarks**: The ovipositor is an egg-laying appendicular organ present in several hexapod groups.

[210] Pygidium [Vetal216]

0. Absent

1. Present

[211] Type of pygidial fusion [Vetal217]

0. Partial

1. Complete

[212] Axial elevation of pygidium [Vetal218]

0. Absent

1. Present

[213] Pygidial ornamentation [Vetal219]

0. Smooth

1. Spinose

**4. Backbone constraint modified from Vannier et al.,** (1).

“Crustacea” as: Argulus+Ostracoda+Triops+Artemia+Copepoda+Cyzicus+Leptostraca+Stomatopoda+Isopoda+Euphasiacea+Cephalocarida+Remipedia+Anurida+Allacma+Zygentoma+Odonata+Mantodea+Hymenoptera+Diptera+Coleoptera

“Chelicerata” as: Pycnogonida+Limulus+Archegozetes+Opiliones+Scorpiones+Araneae

“Myriapoda” as: Chilopoda+Diplopoda+Pauropoda+Symphyla

Constraints

Constraint Node 1, partial: (Coleoptera+Diptera| “Crustacea” + “Chelicerata” + “Myriapoda”)

Constraint Node 2, partial: (Coleoptera+Diptera+Hymenoptera| “Crustacea” + “Chelicerata” + “Myriapoda”)

Constraint Node 3, partial: (Coleoptera+Diptera+Hymenoptera+Mantodea| “Crustacea” + “Chelicerata” + “Myriapoda”)

Constraint Node 4, partial: (Coleoptera+Diptera+Hymenoptera+Mantodea+Odonata| “Crustacea” + “Chelicerata” + “Myriapoda”)

Constraint Node 5, partial: (Coleoptera+Diptera+Hymenoptera+Mantodea+Odonata+Zygentoma| “Crustacea” + “Chelicerata” + “Myriapoda”)

Constraint Node 6, partial: (Anurida+Allacma | “Crustacea” + “Chelicerata” + “Myriapoda”)

Constraint Node 7, partial: (Anurida+Allacma+Coleoptera+Diptera+Hymenoptera+Mantodea+Odonata+Zygentoma | “Crustacea” + “Chelicerata” + “Myriapoda”)

Constraint Node 8, partial: (Copepoda+Leptostraca+Stomatopoda+Isopoda+Euphasiacea | “Crustacea” + “Chelicerata” + “Myriapoda”)

Constraint Node 9, partial: (“Crustacea” | Argulus+Ostracoda + “Chelicerata” + “Myriapoda”)

Constraint Node 10, partial: (“Crustacea” | “Chelicerata” + “Myriapoda”)

Constraint Node 11, partial: (Argulus+Ostracoda | “Crustacea” + “Chelicerata” + “Myriapoda”)

Constraint Node 12, partial: (Diplopoda+Pauropoda | “Crustacea” + “Chelicerata” + “Myriapoda”)

Constraint Node 13, partial: (Diplopoda+Pauropoda+Symphyla | “Crustacea” + “Chelicerata” + “Myriapoda”)

Constraint Node 14, partial: (“Myriapoda”| “Crustacea” + “Chelicerata”)

Constraint Node 15, partial: (“Chelicerata”| “Crustacea” + “Myriapoda”)

Constraint Node 16, partial: (Archegozetes+Opiliones+Scorpiones+Araneae | “Crustacea” + “Chelicerata” + “Myriapoda”)

Constraint Node 17, partial: (Limulus+Archegozetes+Opiliones+Scorpiones+Araneae | “Crustacea” + “Chelicerata” + “Myriapoda”)

**References**

1. Vannier J, Aria C, Taylor RS, Caron JB. *Waptia fieldensis* Walcott, a mandibulate arthropod from the middle Cambrian Burgess Shale. R Soc Open Sci. 2018;5(6)(172206).

2. Yang J, Ortega-Hernández J, Lan T, Hou JB, Zhang XG. A predatory bivalved euarthropod from the Cambrian (Stage 3) Xiaoshiba Lagerstätte, South China. Sci Rep. 2016;6(27709):1–10.

3. Legg DA, Caron JB. New middle Cambrian bivalved arthropods from the Burgess Shale (British Columbia, Canada). Palaeontology. 2014;57(4):691–711.

4. Legg DA, Sutton MD, Edgecombe GD, Caron JB. Cambrian bivalved arthropod reveals origin of arthrodization. Proc R Soc B Biol Sci. 2012;279(1748):4699–704.

5. Briggs DEG. Bivalved arthropods from the Cambrian Burgess Shale of British Columbia. Palaeontology. 1977;20(Part 3):595–621.

6. Zhai D, Ortega-Hernández J, Wolfe JM, Hou X, Cao C, Liu Y. Three-Dimensionally Preserved Appendages in an Early Cambrian Stem-Group Pancrustacean. Curr Biol. 2019;29(1):171-177.e1.

7. Aria C, Caron JB, Gaines R. A large new leanchoiliid from the Burgess Shale and the influence of inapplicable states on stem arthropod phylogeny. Palaeontology. 2015;58(4):629–60.

8. Chen JY, Vannier J, Huang DY. The origin of crustaceans: New evidence from the early Cambrian of China. Proc R Soc B Biol Sci. 2001;268(1482):2181–7.

9. Moysiuk J, Caron JB. Burgess Shale fossils shed light on the agnostid problem. Proc R Soc B Biol Sci. 2019;286(1894).

10. Whittington HB. *Tegopelte gigas*, a second soft-bodied trilobite from the Burgess Shale, middle Cambrian, British Columbia. 1985;59(5):1251–74.

11. Lamsdell JC, Stein M, Selden PA. *Kodymirus* and the case for convergence of raptorial appendages in Cambrian arthropods. Naturwissenschaften. 2013;100(9):811–25.

12. Aria C, Caron JB. Burgess Shale fossils illustrate the origin of the mandibulate body plan. Nature. 2017;545(7652):89–92.

13. Müller KJ, Walossek D. External morphology and larval development of the Upper Cambrian maxillopod *Bredocaris admirabilis*. Foss Strat. 1988;23:70.

14. Shu D, Zhang X. *Kuamaia*, an Early Cambrian predator from the Chengjiang fossil Lagerstätte. J Northwest Univ Spec Vol. 1996;27–33.
